# Ten simple rules for running a successful women-in-STEM organization on an academic campus

**DOI:** 10.1371/journal.pcbi.1007754

**Published:** 2020-05-07

**Authors:** Deborah D. Rupert, Alexandra C. Nowlan, Oliver H. Tam, Molly Gale Hammell

**Affiliations:** 1 Cold Spring Harbor Laboratory, Cold Spring Harbor, New York, United States of America; 2 Stony Brook University, Medical Scientist Training Program, Stony Brook, New York, United States of America; Carnegie Mellon University, UNITED STATES

## Abstract

The current academic culture facing women in science, technology, engineering, and math (STEM) fields in the United States has sparked the formation of grassroots advocacy groups to empower female scientists in training. However, the impact of these initiatives often goes unmeasured and underappreciated. Our Women in Science and Engineering (WiSE) organization serves postdoctoral researchers, graduate students, and research technicians (trainees) at a private research institute for biological sciences. Here we propose the following guidelines for cultivating a successful women-in-STEM-focused group based upon survey results from our own scientific community as well as the experience of our WiSE group leaders. We hope these recommendations can provide guidance to advocacy groups at other research and academic organizations that wish to strengthen their efforts. Whereas our own group specifically focuses on the underrepresented state of women in science, we hope these guidelines may be adapted and applied to groups that advocate for any minority group within the greater scientific community (i.e., those of gender, race/ethnicity, socioeconomic background, sexual orientation, etc.).

## Author summary

Women are underrepresented in leadership positions in the fields of science, technology, engineering, and math (STEM) despite obtaining undergraduate and graduate-level degrees at roughly the same rate as men. To take advantage of this resource of highly trained women in STEM fields, there needs to be better career support for female scientists in training. The authors report on their experience running a women-in-STEM-focused group to foster a more supportive, collaborative, and egalitarian scientific community. In particular, the authors use data-driven metrics to evaluate the success of their group in meeting the needs of their community. The results of this evaluation are presented as a set of 10 guidelines for establishing and maintaining advocacy groups that support the careers of women in science.

## Introduction

Substantial data show that although the number of undergraduate and graduate degrees in STEM awarded to women is roughly equal to the number awarded to men, women remain underrepresented in professional leadership positions in both academia and industry [1–3]. Compared with men, women are more likely to be targets of hiring bias, microaggressions, and sexual harassment and receive fewer invitations to publish and present their research [3–5]. These factors have direct consequences on career outcomes and long-term retention of women in STEM fields [6]. Indeed, not just overt bias but ambivalence toward sexism and bias has been reported to negatively affect female trainees [7]. The marginalization of women in STEM was publicly recognized in a landmark study conducted at Massachusetts Institute of Technology (MIT) in the late 1990s [8]. Many of the identified inequities have persisted, leaving female scientists dissatisfied with the limited extent of reform within academic institutions and STEM communities more generally. Moreover, these inequities negatively affect the scientific community at large; driving female talent out of science restricts scientific progress and has larger consequences for the health of the general population when medically relevant research at both the bench and public health levels are gender restrictive.

In recent years, there has been considerable investment in initiatives to support women’s advancement in STEM and spark change from the bottom up [9]. These grassroots organizations are critical for empowering female scientists in training. At Cold Spring Harbor Laboratory (CSHL), a private institute for biological research and education, our Women in Science and Engineering (WiSE) organization is one such group created by and for trainees: postdoctoral researchers, graduate students, and research technicians. Our aim is to foster a more supportive, collaborative, and equal-minded scientific community by providing a platform for professional development, education, and empowerment. Over the past 4 years, we have refined our goals and activities to more effectively promote our mission and support our members. Our organization has been successful in establishing institutional support and hosting well-attended, lab-wide events. According to anonymous community feedback, approximately 80% of survey participants campus-wide considered CSHL’s WiSE program to be moderately or very successful based on their understanding of our organization’s mission and goals.

Examining this feedback has allowed us to evaluate our group’s accomplishments and shortcomings. We use the conclusions about these strengths and weakness as well as our own experience to propose the following “rules” for women-in-STEM advocacy groups that are starting up or who wish to bolster their own efforts.

## Rule 1: Define and communicate the goal(s) of the group

Assessment of an organization’s success is best measured against a clear mission statement. We suggest formalizing both broad and event-specific goals in writing and actively revisiting these pieces of writing to adapt them as the group evolves—i.e., annually when new leadership is elected. For example, our group’s general mission statement is as follows:

“To build a more supportive, collaborative, and equal scientific community for all. We provide a platform for professional development and empowerment through mentorship, career planning, and educational opportunities tailored toward issues disproportionately affecting women.”

But the “mission” for a specific event might read as:

“The WiSE Retreat is an annual, day-long meeting that welcomes scientists-in-training at CSHL to attend lectures on gender disparity. The goals of the day are to: 1. familiarize the WiSE group and our greater community with gender disparity literature; 2. encourage the application of scientific scrutiny to this body of literature; 3. stimulate continued self-education on this topic.”

Our group broadcasts its goals and announces WiSE-hosted, on- and off- campus events through a website platform (www.cshlwise.org), Twitter account (@CSHLWISE), Instagram (@WISE_CSHL), Facebook (/WISECSHL), and campus-specific Slack account.

Advocacy groups (i.e., institutional initiatives or student-run groups that aim to promote equity for scientists of minority status) should be cognizant that their broadcasts will suffer from self-selection bias on the part of followers; thus, some subpopulations of the academic campus will be better informed than others. We encourage fellow organizations to evaluate their communication strategies, examine when they fall short, and determine how to address audiences that may be less familiar with the group.

## Rule 2: Carefully structure the organization

Our WiSE group addresses the present needs of the CSHL community through five main “branches” (subcommittees): Institutional Initiatives, Professional Development, In-House Education, Mental Health, and Outreach. Each branch reaches toward an individual goal (Table 1), whereas all five branches work together to support female researchers on our campus in reaching the height of their potential.

**Table 1:** List of events hosted by our group to date, broken down by category.

Branch of Our Organization	Goal	Example Events
Institutional initiatives	Highlight the accomplishments of prominent female scientists in biology and provide networking opportunities for our trainees	McClintock campus-wide lectures
		Women in Biology Speakers List
		WiSE Mentorship awards
		Breakfast with invited female speakers
Professional development	Promote the professional growth of our trainees	Negotiation workshop
		Financial management workshop
		Presentation skills workshops
		Practice talks for graduate students and postdocs
Outreach	Grow the next generation of scientists, feminists, and their supporters committed to equality in STEM	Girl Scouts Brain Awareness Week
		Basic coding bootcamp
		Summer camp
		Wiki-edit-athon
		Science cafes in high schools
Education	Systematically review data-driven literature examining gender disparities in STEM	Journal clubs
		Annual education retreat
Mental health	Combat the mental health crisis endured by trainees, which disproportionately affects women	Open mic mental health night

Events reflect the pillars of our organization: institutional initiatives, professional development, outreach, education, and mental health. To learn more about these events, please visit our website: cshlwise.org

Abbreviation: STEM, science, technology, engineering, and math

We encourage our counterparts at other academic institutions to carefully consider their primary goals and how to best go about accomplishing these, given the limitations of their women-power, resources, and institutional support. Our own group was originally established with three of its now five branches, which have been modified as the group’s leadership evolves (see Rule 3).

We further recommend careful consideration of how the structure of the organization can best benefit its members. Leaders should have realistic conversations about board member expectations when inviting new members to take on leadership roles; for example, we ask board members to commit to a position for 1–2 years, and certain roles (i.e., president) can only be held by a board member with prior experience in a different position. At the same time, being able to adapt board roles to fit the time commitments and strengths of future leaders is critical for achieving realistic progress. It is important to remember that our members are scientists first and foremost, and ideally, their level of involvement in advocacy should be balanced such that it does not interfere with their research progress.

**Fig 1:**
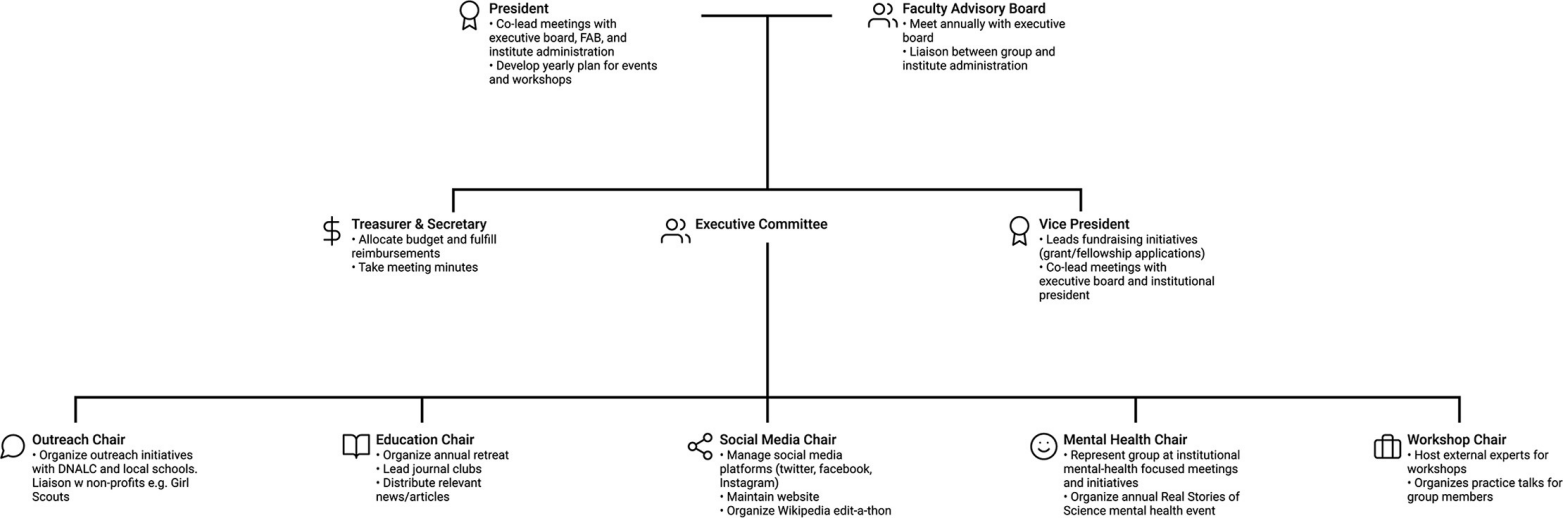
Schematic of WiSE organizational leadership. WiSE is a trainee-led group with students, postdoctoral fellows, and technicians filling all of the leadership roles outside of the FAB. WiSE members are encouraged to volunteer and participate in the events organized by the executive committee chairs that oversee the subcommittees/branches detailed above. Active members typically go on to assume a role on the board; this allows for many leadership opportunities for students and ensures that the group remains active as responsibilities are handed off to subsequent group leaders. DNALC, DNA Learning Center; FAB, Faculty Advisory Board; WiSE, Women in Science and Engineering.

Our group’s leadership is structured to promote gradual increases in responsibilities (Fig 1). Leaders develop and practice critical skills such as project management, communication, and professionalism through their work in WiSE. When senior leadership members transition to the next stage of their training, their roles and responsibilities must be handed off to the next leader with clear expectations and an open-door policy. Mentoring within the group is essential so that senior leaders can pass the torch on to younger members. This benefits both parties, allowing younger members to acquire more responsibility and allowing senior members to release that responsibility when it becomes too demanding in conjunction with their career demands. Volunteer advocacy gives scientific trainees real-life experience with leadership, time management, and negotiation, all of which are integral to a career in science [10]. Thus, we argue that grassroots organizations like our own are a fundamental part of trainee development in addition to the scientific development they undergo.

## Rule 3: Harness the strengths of members

Our group is composed of women and allies of women in STEM, at various levels of scientific training (technicians, graduate students, and postdocs) and across various fields of biological sciences (cancer biology, neuroscience, genomics, etc.). We see strength in our ability to recognize members as having unique backgrounds, interests, and skill sets (scientific and otherwise) and harness this diversity. This is reflected in our hosted events (Table 1). For example, members with expertise in computer programming have led hands-on, basic coding bootcamps for an audience of middle-school-aged young women. A member with a proclivity for social media organized a “How To” wiki-edit-a-thon to help expand digital media articles on prominent female scientists. Members with an interest in teaching careers have applied and expanded upon their prior experience through our educational outreach events. The various interests of our members are also reflected in the organization of our subcommittees. For example, our in-house education branch was added to distinguish our external outreach events from our efforts to educate our own community. The addition of a branch is a large undertaking that must be carefully considered by the board and backed by an appropriate level of commitment by the founding members. Similarly, when the group’s efforts become spread too thin, removal of specific events or branches must be considered to maintain the integrity of the group’s efforts.

We encourage advocacy groups to balance the specific interests of active members with the assurance that these events meet the expectations of the target audience. At the same time, recognizing that targeted audience subpopulations vary by event is fundamental for allowing specialization of one’s initiatives and breadth of the organization’s reach as a whole.

## Rule 4: Identify goals common to institutions and groups

The degree to which an institute recognizes or resists gender disparity issues undoubtedly varies. Despite this, common objectives between women-in-STEM-focused groups and their institutions can help benefit both parties. For example, institutions are self-motivated to attract strong scientific minds and “big name” guest lecturers. WiSE groups should be mindful of the goals of their underlying institution, the decision-making leadership and stakeholders within that institution, and the strength of other groups that it chooses to collaborate with. Impact can best be achieved where all parties gain. For example, our WiSE organization has negotiated for institutional support (both financial and logistic) to host two prominent female scientists to give guest lectures through the McClintock Lecture series, in which we honor the legacy of Nobel laureate and CSHL scientist Barbara McClintock (see Table 1). Understanding that academically interesting events can be used as a draw to pull in a wider audience can be a powerful way to share the message of women-in-STEM groups beyond those who would regularly be exposed to it. As such, the McClintock lectures, which draw widely from the CSHL community, provide a platform for promoting the WiSE group and announcing other upcoming WiSE events.

We further highlight the value that advocacy groups add to their institutions. Trainees expect this type of community support on campus and look to the leadership to endorse this overdue shift in academic culture toward supportive and inclusive environments [1,2,9]. Members of our group are frequently called upon by the institute in matters of public relations to represent our goals as well as those of the larger institution. Accepting these opportunities whenever possible is paramount to cultivating a productive relationship with the administration.

## Rule 5: Promote diversity within the group

We believe that attracting diversity to one’s group in terms of gender, position, and racial/ethnic background is critical for the success of the organization. We have found that WiSE groups, our own included, often become tunnel-visioned and fail to engage untapped sources of support. For example, staff personnel on our campus pointed out the lack of roles for them to play in the group; this is a large body of supporters whose assistance remains underappreciated and untapped.

We further encourage collaboration with specific diversity advocacy groups to address issues that are common for racial and gender minorities within STEM, i.e., intersectionality. For example, we have organized an allyship workshop with CSHL’s Diversity Initiative for the Advancement in STEM (DIAS). Commonalities between this group and our own allow us to build stronger coalitions that can work together toward larger goals.

Understanding how to cater to the needs of each group is fundamental for garnering support from the diverse populations that make up any modern academic environment.

## Rule 6: Cultivate mentorship

Mentorship is fundamental for the development and retention of female students in STEM [11,12]. Identifying strong faculty mentors can help WiSE groups achieve better communication between the group and the administration. Our WiSE Faculty Advisory Board consists of active CSHL research faculty who have demonstrated their commitment to the WiSE mission, advocating for the inception of the group, participation and advocacy for gender studies research, and participation in WiSE events. The primary responsibility of the Faculty Board is to provide mentorship and guidance to WiSE members (the full description of roles and responsibility of the Faculty Board can be found on our website: http://cshlwise.org/about/leadership/).

We encourage other groups to make clear the “what” and “how” of mentorship [13–15]. Our Faculty Board consists of a mixture of male and female scientists as well as assistant, associate, and full professors. The diversity of this advisory board is an important factor for its success. For example, selection of male mentors enhances the inclusivity of the group to nonfemale supporters and reinforces the message that WiSE is open to all members of the community.

That said, we remind fellow grassroots organizations that faculty members are there to guide and assist but not to lead the student groups. The responsibility of leadership and decision-making should rest in the hands of student leaders. This allows trainees to gain leadership experience while keeping the focus of our organization on issues most strongly affecting our members.

## Rule 7: Reach out beyond your scientific community

The ability to see role models in science from a diverse array of backgrounds, especially at an early age, can impact implicit biases widely. Although presenting female role models for younger generations has positive impact [15], after several years of doing so we have realized the need to expand these efforts to educate parents, teachers, and community leaders and to develop community contacts/connections for future events. We encourage groups like our own to assess their areas of expertise and consider creative ways to engage their local communities beyond their academic campuses. For example, to include older populations in our education initiatives, we have invited students to bring their parents to our lectures focused on genetically modified organisms (GMOs), with a question-and-answer panel of plant biologists. Such efforts reflect well on the home institution and may provide an important source of independent funding for future initiatives (see Rule 9).

## Rule 8: Assess, analyze, adapt

In an effort to strengthen our own group, we have gathered, analyzed, and discussed the survey data that led to the conclusions drawn here. We hope doing so will serve as an example for other groups to apply their critical, scientific nature toward their passion projects. Whenever possible, we encourage groups to send out follow-up surveys to assess the strengths and weaknesses of specific events; use the SMART (specific, measurable, achievable, relevant, and time-based) goal technique to ensure follow-through. Discuss as a team the cost-benefit analysis of hosting each event in to order to determine where limited time, effort, and resources are best spent.

## Rule 9: Identify your external resources

There are a growing number of resources to aid grassroots organizations like our own. We encourage groups to familiarize their leadership and members with the currently available online resources from government institutes, academic centers, and private organizations. These tools range from concepts for workshops to data-based reports of gender bias, to funding opportunities. For example, we have found that applications to small, community-based-initiative grants may be more approachable for grassroots organizations like our own as opposed to larger, national-based opportunities. We have had success in such smaller applications but caution other groups that all grant applications are competitive and time-demanding, especially when diversity advocacy is a “passion” project alongside full-time scientific demands.

We provide a more extensive, centralized list of available resources on our website (http://cshlwise.org/resources) but recommend the following as examples: Harvard’s Implicit Bias Training; “The Sexual Harassment of Women: Climate, Culture, and Consequences in Academic sciences, Engineering, and Medicine” published by The National Academies of Sciences; and resources offered on the HeForShe website (https://www.heforshe.org/en) [5,16,17].

In addition, we recommend following and interacting with similar groups via social media (e.g., academic Twitter—for example, @500womensci, @MeTooSTEM, etc.). Our own group can be found on Twitter at @CSHL_WISE.

## Rule 10: Use criticism constructively

We acknowledge the great amount of time and effort required to successfully organize a WiSE group. Addressing criticism in the face of this effort is challenging but nevertheless valuable. Anonymous feedback, including critiques from those who generally support the goals of the group, should be expected. In addition to this, we want other advocacy leaders to be prepared to face criticism from those who may not currently share the goals of the group but who are still part of our community. For example,

“WiSE has created a perception that female graduate students do not want to work and they… blame men when they do not have data or [are] not productive in the lab.”

We use this as an opportunity to demonstrate our last point: above all else, persist. Encouraging others to embrace diversity and inclusivity in science takes time and work but is an important part of improving science as a field. Using critiques like the one above to inspire rather than discourage our work, we continue to modify and amend our group’s focus and events in a continual effort to improve our service and to support the women on our academic campus.

## Conclusion

Here we have shared lessons learned on growing a grassroots organization, targeting specific inequities while balancing inclusivity, and accepting constructive criticism. Our recommendations are by no means a one-size-fits-all model. However, we hope that advocacy groups at all stages are encouraged to conduct similar data-driven evaluations of their efforts and take advantage of our findings at their home institutes so that the STEMinist community at large works resourcefully toward the greater goal of achieving gender parity in STEM.

A longer version of this manuscript, which includes the WiSE Survey and analysis of the survey data, is available at the following DOI on BioRxiv.org for those interested in more details about the WiSE group and the methods used for self-evaluation: https://doi.org/10.1101/2020.02.20.958629.
